# Declined plasma microfibrillar-associated protein 4 levels in acute coronary syndrome

**DOI:** 10.1186/s40001-023-01002-z

**Published:** 2023-01-18

**Authors:** Chunming Han, Yuanshu Peng, Xiaoyan Yang, Zongsheng Guo, Xinchun Yang, Pixiong Su, Shubin Guo, Lei Zhao

**Affiliations:** 1 Emergency Medicine Clinical Research Center, Beijing Chao-Yang Hospital, Capital Medical University, Beijing, China; 2 Beijing Key Laboratory of Cardiopulmonary Cerebral Resuscitation, Beijing, 100020 China; 3 Heart Center, Beijing Chao-Yang Hospital, Capital Medical University, Beijing, 100020 China

**Keywords:** Microfibrillar-associated protein 4, Coronary stenosis, Acute coronary syndrome, Myocardial infarction, Biomarker

## Abstract

**Background:**

Microfibrillar-associated protein (MFAP4), initially identified as an extracellular matrix protein, has been demonstrated in multiple human disorders, but it is yet to be discovered following acute coronary syndrome (ACS) in clinical practice. Therefore, this study aimed to investigate the relationship between circulating MFAP4 levels and coronary stenosis in ACS.

**Methods:**

We performed the study in 148 ACS subjects, including 75 ST-segment elevation myocardial infarction (STEMI), 27 non-ST-segment elevation myocardial infarction (non-STEMI) and 46 unstable angina (UA). Clinical variables were collected and Gensini and Syntax stenosis scoring systems were applied to assess the severity of coronary stenosis. Kaplan–Meier and logistic regression analysis were used to analyze the relationship between MFAP4 and the severity of coronary stenosis or ACS outcomes. Spearman analysis was used to describe the correlation between MFAP4 and clinical parameters.

**Results:**

Circulating MFAP4 levels were significantly decreased in the STEMI group (0.008 ng/ml) compared with the non-STEMI group (0.014 ng/ml) and UA group (0.019 ng/ml) (*p* < 0.001). After adjusting for confounding factors, we found that MFAP4 was an independent risk factor for STEMI (odds ratio = 0.395, 95% CI 0.174–0.895, *p* = 0.026). MFAP4 level was negatively correlated with Gensini score and Syntax score (*r* = − 0.311 and − 0.211, *p* < 0.001 and 0.01, respectively). Based on the MFAP4 level of 0.117 ng/ml, ACS patients were divided into two groups: the low-MFAP4 group (< 0.117 ng/ml, *n* = 60) and the high-MFAP4 group (≥ 0.117 ng/ml, *n* = 88). After the median follow-up of 165 days, Kaplan–Meier survival analysis revealed that the MACE-free rate was significantly lower in ACS patients with lower MFAP4 levels (*p* = 0.009).

**Conclusions:**

MFAP4 has a potential as a biomarker for the degree of coronary stenosis in ACS. Confirmation of observations in larger cohorts and longer follow-up periods is warranted.

**Supplementary Information:**

The online version contains supplementary material available at 10.1186/s40001-023-01002-z.

## Introduction

Acute coronary syndrome (ACS) is the most common cardiovascular emergency, accounting for nearly half of the global cardiovascular morbidity and mortality, placing an enormous economic burden worldwide [1]. Typically, depending on the range of myocardial ischemia, ACS may present as unstable angina (UA), non-ST-segment elevation myocardial infarction (non-STEMI), and ST-segment elevation myocardial infarction (STEMI). Since ACS may proceed into irreversible myocardial damage, early and precise identification of such urgent situation are crucial. So far, detection of coronary artery stenosis heavily relies on invasive methods, such as coronary angiography. Although there are some biomarkers in identifying myocardial necrosis, including cardiac troponins (cTn) and creatine kinase-MB (CKMB)—the gold standard markers, they cannot reflect the severity of coronary stenosis, since patients in severe vascular condition may need individualized treatment. Thus, it is significant to find novel biomarkers that precisely detect myocardial damage and also reflect the severity of coronary stenosis.

Microfibrillar-associated protein 4 (MFAP4), also named microfibrillar-associated glycoprotein 4 (MAGP4), known as 36-kDa microfibril-associated glycoprotein, is a secreted matricellular protein and belongs to the fibrinogen-related domain superfamily, including fibroleukin, ficolin, angiopoietins, and tenascin, which is initially associated with tissue homeostasis, cardiovascular system development and normal endothelial function [2]. MFAP4, mainly located in elastic fibers and highly expressed in blood vessels, probably contributed to vascular remodeling. Besides large arteries and arterioles, MFAP4 mRNA transcript levels were relatively higher in myocardial tissue and lung [3], which is in agreement with its important role in cardiac hypertrophy, asthma and chronic obstructive pulmonary disease [4]. Recently, animal studies have reported that MFAP4 deficiency inhibited myocardial hypertrophy and myocardial fibrosis, and improved cardiac function [5–7]. Notably, some reports even suggested the possibility of using MFAP4 as a biomarker in atherosclerosis-related diseases [3]. Additionally, MFAP4 has been identified with a lower heritability and relatively limited basic variation, implying MFAP4 as an excellent biomarker candidate in reflecting the process of disease induction [8, 9]. And, the association between plasma MFAP4 and other clinical parameters and its role in the prognosis of ACS is still under discussion. Accordingly, in this study, we aimed to investigate the correlation between severity of coronary stenosis, calculated by coronary angiography, and the circulating MFAP4 levels in a cohort of patients admitted with ACS.

## Materials and methods

### Study population

**Fig. 1:**
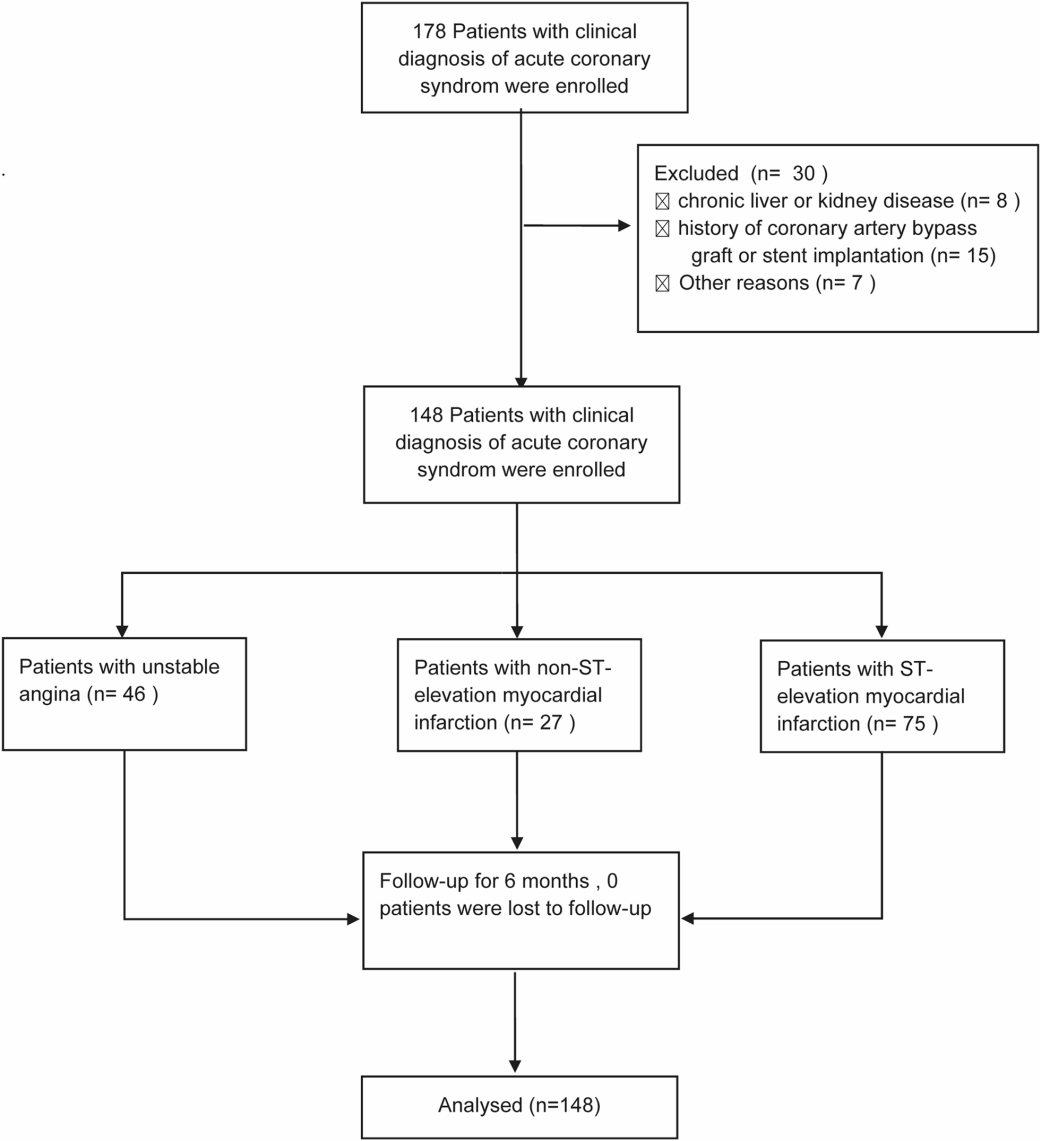
Flowchart of patient enrollment

In this study, 148 ACS patients, including 75 STEMI, 27 non-STEMI and 46 UA, admitted at the Department of Cardiology, Beijing Chaoyang Hospital from May 2021 to July 2021 were recruited in this study (Fig. 1). Clinical classification of ACS was based on expert consensus statements. All subjects with a history of stent implantation, a history of coronary artery bypass graft, cardiomyopathy, myocarditis, congenital heart disease, chronic liver or kidney disease, systemic or local inflammatory or infectious disease, autoimmune disease, pregnancy, and malignancy were excluded from the study. Written, informed consent was obtained from all subjects before their participation in the study. The study protocol was approved by the Beijing Chaoyang Hospital Ethics Committee and adhered to the Declaration of Helsinki. This study was registered in the Chinese Clinical Trial Registry (No. ChiCTR2100045902).

### Laboratory assay

Venous blood samples were drawn in tubes with ethylenediaminetetraacetic acid (EDTA) and centrifuged at 3000 rpm for 10 min at 4 °C. Plasma was stored in a – 80 ℃ ultra-low-temperature refrigerator until biochemical analysis. Plasma MFAP4 levels were measured using commercially available microplate ELISA kit (Human Microfibrillar Associated Protein 4 ELISA kit, Abbexa LTD, Cambridge, UK) following the sandwich ELISA principle (Catalog No. abx152321, sensitivity < 0.121 ng/mL, intra-assay coefficient of variation < 10%, and inter-assay coefficient of variation < 10%). Other clinical and biochemical parameters were measured by standard procedures.

### Evaluation of coronary artery disease severity

Coronary angiographic image was collected from the catheter laboratory during hospitalization, and Gensini and Syntax stenosis scoring systems were applied to assess the severity of coronary stenosis by two independent experienced interventional clinicians. As published previously, the Gensini score was based on the grade of stenosis, and the sum of the scores for individual lesions was defined the severity of coronary artery disease. The Syntax score was an anatomical calculator that quantitatively describes vessels with diameters > 1.5 mm and stenosis > 50% in the light of location, function, complexity and obstruction, and was performed with internet-based SYNTAX calculator (www.syntax.score.com).

### Statistical analysis

Normal distribution of data was assessed using the Kolmogorov–Smirnov test. Numerical variables with a normal distribution were presented as the mean ± SE, numerical variables with a skewed distribution as the median (P25, P75). The *χ*^2^ test was performed to analyze demographic data. Comparisons in groups with parametric conditions were performed using Student’s *t* test, and with the Mann–Whitney *U* test in groups without parametric conditions. Subsequently, analysis was undertaken through multivariate, step-wise forward conditional logistic regression analysis to assess a set of independent variable predictors of ACS. Spearman correlation was used for correlation analyses. The sensitivity and specificity of the MFAP4 level were determined by the receiver operating characteristic curve (ROC), and the best cut-off value was set to maximize the sensitivity and minimize1–specificity. Kaplan–Meier survival curves were applied to depict survival curves and a log-rank test was used to assess survival differences between groups. Clinical events were followed up since patient’s hospitalization, and after discharge medical record and phone interviews help record major adverse coronary events (MACE), including cardiovascular death, rehospitalization, advanced heart failure and revascularization. Values of *p* < 0.05 were regarded as statistically significant. All statistical analyses were performed with IBM SPSS Statistics 24.0 (IBM Corp, Armonk, NY).

## Results

### Baseline clinical characteristics of the cohort

**Fig. 2:**
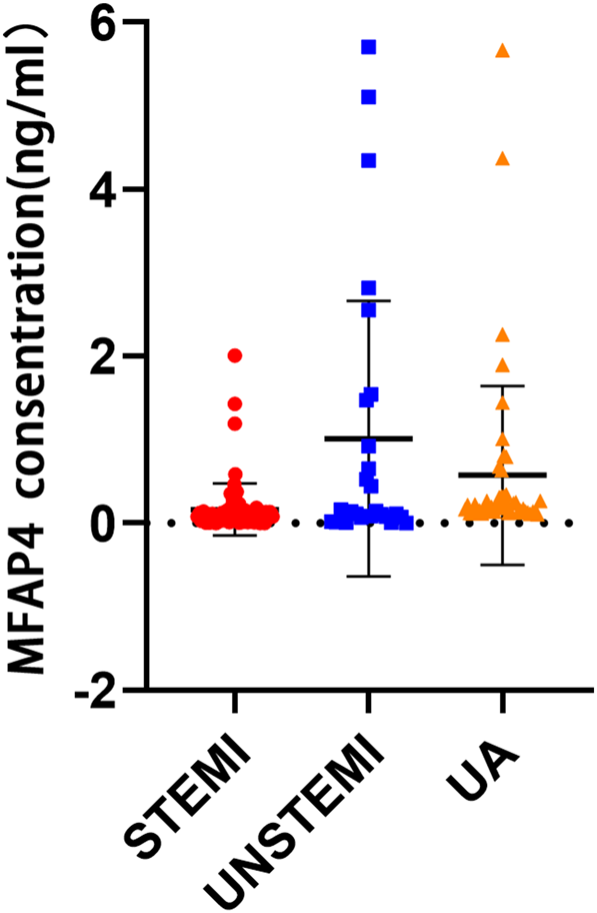
MFAP4 levels in different groups. MFAP4 levels were significantly decreased in the STEMI group (0.008 ng/ml) compared with the UNSTEMI group (0.014 ng/ml) and UA group (0.019 ng/ml) (*p* < 0.001)

The present study included ACS patients with 75 STEMI, 27 non-STEMI and 46 UA. The baseline clinical characteristics are described in Table 1. The mean age of the study population was 61 ± 11 years and 118 (79.7%) participants were male. Regarding demographic data, except for hypertension, which was more common in UA patients, there was no other statistically significant difference regarding age, gender, prevalence of diabetes, hyperlipidemia and arrhythmia among three groups. In the laboratory measurement, with gradually aggravated cardiac function and increased myocardial injury, ACS patients probably faced unregulated abnormal lipid and glucose metabolism, increased inflammatory biomarkers: higher leukocytes (*p* < 0.001) and higher C-reactive protein (*p* < 0.001), and accompanied with more renal and hepatic dysfunction (increased creatinine, alanine aminotransferase and aspartate aminotransferase). As shown in Fig. 2 and Table 1, the median MFAP4 levels were 0.133 ng/ml, with an interquartile range of 0.065–0.262 ng/ml. Remarkably, circulating MFAP4 levels were gradually decreasing following the developed degree of myocardial ischemia, which were lowest in the STEMI group (STEMI vs. non-STEMI *p* = 0.018 and STEMI vs. UA *p* < 0.001, respectively). Medication on admission and medication on discharge is shown in Table S1.

**Table 1:** Baseline characteristic of the study population

Clinical characteristics	STEMI	UNSTEMI	UA	P-value
Total *n* = 148	*n* = 75	*n* = 27	*n* = 46	
Age [years]	60.89 ± 12.16	60.44 ± 10.16	61.72 ± 8.66	0.759
Male [*n* (%)]	62 (82.7)	20 (74.1)	36 (78.3)	0.608
Smoker [*n* (%)]	48 (64)	17 (63.0)	30 (65.2)	0.446
Drinker [*n* (%)]	18 (24)	9 (33.3)	15 (32.6)	0.446
Hypertension [*n* (%)]	40 (53.3)	16 (59.3)	36 (78.3)	0.022
Diabetes mellitus [*n* (%)]	27 (36.0)	12 (44.4)	15 (32.6)	0.362
Hyperlipidaemia [*n* (%)]	73 (97.3)	24 (88.9)	45 (97.8)	0.12
Cardiac arrhythmia [*n* (%)]	13 (17.3)	5 (18.5)	3 (6.5)	0.197
Old cerebral infarction [*n* (%)]	7 (9.5)	3 (11.1)	6 (13.0)	0.828
Length of hospital stay [days]	8 (7, 12)	7 (5, 9)	4 (3, 5)	< 0.001
SBP [mmHg]	127.41 ± 14.68	125.70 ± 15.98	140.35 ± 10.02	0.001
DBP [mmHg]	75.21 ± 11.82	75.00 ± 11.29	80.26 ± 10.03	0.022
HR [bpm]	78.91 ± 13.33	78.48 ± 11.31	75.72 ± 12.34	0.339
LVEF [%]	57.74 ± 8.27	60.93 ± 8.22	67.41 ± 5.46	< 0.001
WBC [10^9^/L]	9.25 ± 3.29	9.00 ± 3.23	6.98 ± 1.59	< 0.001
Hb [g/L]	134.16 ± 15.71	128.96 ± 14.37	136.83 ± 11.89	0.949
PLT [10^9^/L]	214.57 ± 60.95	210.44 ± 56.07	213.22 ± 50.67	0.955
Cr [μmol/L]	75.19 ± 15.75	69.29 ± 13.92	65.85 ± 14.73	0.007
ALB [g/L]	42.45 ± 3.99	42.67 ± 3.79	42.24 ± 2.94	0.885
AST [U/L]	73 (32, 142)	32 (21, 67)	20 (17, 24)	< 0.001
ALT [U/L]	35 (25, 57)	27(16, 33)	22.5 (16.75, 27.25)	< 0.001
BUN [mmol/L]	5.64 (4.86, 7.05)	5.13 (4.37, 6.88)	5.37 (4.68)	0.161
Na [mmol/L]	138.5 (136.9, 140.5)	139.3 (137.1, 140.5)	140.25 (139.20,141.03)	0.002
K [mmol/L]	4 (3.8, 4.3)	4 (3.8, 4.3)	4 (3.8, 4.2)	0.989
HbA1C [%]	6.2 (5.7, 7.3)	6.3 (5.875, 8.025)	6.05(5.6, 7)	0.486
TC [mmol/L]	4.88 (4.31, 5.43)	5.05 (3.92, 5.68)	3.265 (2.87, 3.79)	< 0.001
LDL [mmol/L]	3.45 (2.79, 3.94)	3.48 (2.95, 4.04)	1.935 (1.408, 2.36)	< 0.001
TG [mmol/L]	1.35 (0.99, 1.79)	1.93 (1.09, 3.23)	1.09 (0.798, 1.708)	0.008
Lp (a) [mg/dl]	18.9 (8.8, 30.6)	16.1 (8, 57.2)	14.25 (8.525, 33.35)	0.864
Glucose [mmol/L]	7.93 (6.64, 10.53)	7.1 (6.07, 9.38)	5.015 (4.523, 6.49)	< 0.001
BNP [pg/ml]	201 (105.75, 290.75)	151 (57, 294.5)	42 (12, 67.75)	< 0.001
CK-MB [ng/ml]	8.9 (2.7, 43)	2.9 (1, 11.8)	1.1 (0.575, 1.525)	< 0.001
cTNI [ng/ml]	3.18 (4.49, 81.71)	8.53 (1.98, 34.84)	0 (0, 0.03)	< 0.001
hs-CRP [mg/L]	5.09 (2.17, 19.52)	15.49 (3.93, 34.69)	1.33 (0.55, 2.89)	< 0.001
MFAP4 [ng/ml]	0.08 (0.03, 0.15)	0.14 (0.07, 1.47)	0.19 (0.14, 0.33)	< 0.001
Gensini score	81 (50, 104)	76 (48, 92)	30 (15.75, 65.25)	< 0.001
Syntax score	24.5 (16, 32.5)	24 (15.5, 30)	17.5 (10, 30.625)	0.104

Data are presented as median (interquartile range) or mean value ± SE

The *p* value reflected the significant difference among the three groups

*SBP* systolic blood pressure, *DBP* diastolic blood pressure, *HR* heart rate, *LVEF* left ventricular ejection fraction, *WBC* white blood cell count, *Hb* hemoglobin, *PLT* platelet, *Cr* serum creatinine concentrations, *ALB* albumin, *ALT* alanine aminotransferase, *AST* aspartate aminotransferase, *BUN* blood urea nitrogen, *HbA1c* blood glycated hemoglobin A, *TC* total cholesterol, *TG* triglyceride, *LDL* low-density lipoprotein cholesterol, *Lp(a)* lipoprotein(a), *BNP* brain natriuretic peptide, *cTNI* cardiac troponin, *CKMB* creatine kinase MB, *hsCRP* high sensitivity C-reactive protein, *MFAP4* microfibrillar-associated protein 4

### Association between plasma MFAP4 levels and biochemical variables or coronary stenosis

The relationship between baseline MFAP4 and other clinical biomarkers were analyzed with spearman correlation models. As shown in Table 2, MFAP4 concentrations correlated negatively with total cholesterol (TC) (*r* = − 0.360, *p* < 0.001), low-density lipoprotein cholesterol (LDL) (*r* = − 0.426, *p* < 0.001), cardiac troponin (cTnI) (*r* = − 0.390, *p* < 0.001), creatine kinase-MB (CK-MB) (*r* = − 0.329, *p* < 0.001), brain natriuretic peptide(*r* = − 0.316, *p* = 0.001), alanine aminotransferase (*r* = − 0.352, *p* < 0.001), aspartate aminotransferase (*r* = − 0.403, *p* < 0.001) and high sensitivity creative protein (*r* = − 0.311, *p* = 0.001), while MFAP4 levels was positively related with left ventricular ejection fraction (*r* = 0.377, *p* < 0.001). Specially, MFAP4 was in negative relationship with Gensini and Syntax stenosis scoring (*r* = − 0.311 and − 0.211, *p* < 0.001 and 0.01, respectively), which present the severity of coronary stenosis.

**Table 2:** Spearman correlation analysis of MFAP4 and clinical parameters

Parameter	MFAP4 in total (*n* = 148)	
	*r*	*p*-value
Diagnosis	0.474	< 0.001
Gensini score	− 0.311	< 0.001
Syntax score	− 0.211	0.010
Male [*n* (%)]	0.016	0.848
Age [years]	0.046	0.582
Length of hospital stay [days]	− 0.443	< 0.001
MACE [days]	− 0.081	0.345
HbA1C [%]	− 0.052	0.536
TC [mmol/L]	− 0.360	< 0.001
TG [mmol/L]	− 0.029	0.727
LDL [mmol/L]	− 0.426	< 0.001
LVEF [%]	0.377	< 0.001
cTNI [ng/ml]	− 0.390	< 0.001
CK-MB [ng/ml]	− 0.329	< 0.001
BNP [pg/ml]	− 0.316	0.001
AST [U/L]	− 0.403	< 0.001
ALT [U/L]	− 0.352	< 0.001
hs-CRP [mg/L]	− 0.311	0.001

*MACE* major adverse coronary events

Other abbreviations are the same as Table 1

The association between ACS and circulating MFAP4 levels was analyzed in logistic regression models. Although MFAP4 did not confer a role in myocardial infarction (MI) (STEMI + non-STEMI) (*p* = 0.304), in STEMI patients, declined plasma MFAP4 levels were more common (OR = 0.216, 95% CI 0.070–0.665, *p* = 0.008, Table 3). When all significant univariate determinants (*p* < 0.05), including conventional coronary artery disease risk factors like TC, LDL, cTnI, CKMB, were entered into multivariate model, decreased MFAP4 concentration was also an independent determinant of STEMI (OR = 0.395, 95% CI 0.174–0.895, *p* = 0.026).

**Table 3:** Logistic regression models

Variable	Univariate analysis		Multivariate analysis	
	OR (95% CI)	*p* value	OR (95% CI)	*p* value
Gender [*n* (%)]	1.603 (0.308–1.549)	0.365		
Age [years]	1.239 (0.967–1.027)	0.821		
Hypertension [*n* (%)]	1.667 (0.234–0.911)	0.064		
Diabetes mellitus [*n* (%)]	1.043 (0.491–1.872)	0.837		
LVEF [%]	0.882 (0.837–0.930)	< 0.001	0.925 (0.872–0.980)	0.008
ALT [U/L]	1.038 (1.018–1.059)	< 0.001		
AST [U/L]	1.017 (1.009–1.026)	< 0.001	1.009 (1.002–1.017)	0.009
BNP [pg/ml]	1.002 (0.999–1.004)	0.162		
CKMB [ng/ml]	1.024 (1.006–1.042)	0.007		
cTNI [ng/ml]	1.019 (1.007–1.030)	0.001		
TC [mmol/L]	1.991 (1.445–2.742)	< 0.001		
LDL [mmol/L]	2.468 (1.719–3.544)	< 0.001	1.806 (1.175–2.777)	0.007
TG [mmol/L]	0.833 (0.620–1.119)	0.225		
MFAP4 [ng/ml]	0.216 (0.070–0.665)	0.008	0.395 (0.174–0.895)	0.026

Abbreviations are the same as Table 1

Adjusted confounders: gender, age, PLT, LVEF, AST, ALT, BNP, CKMB, cTNI, TC, LDL, TG, hypertension, diabetes mellitus, MFAP4

*CI* confidence interval

### Diagnostic and prognostic value of circulating MFAP4 levels

**Fig. 3:**
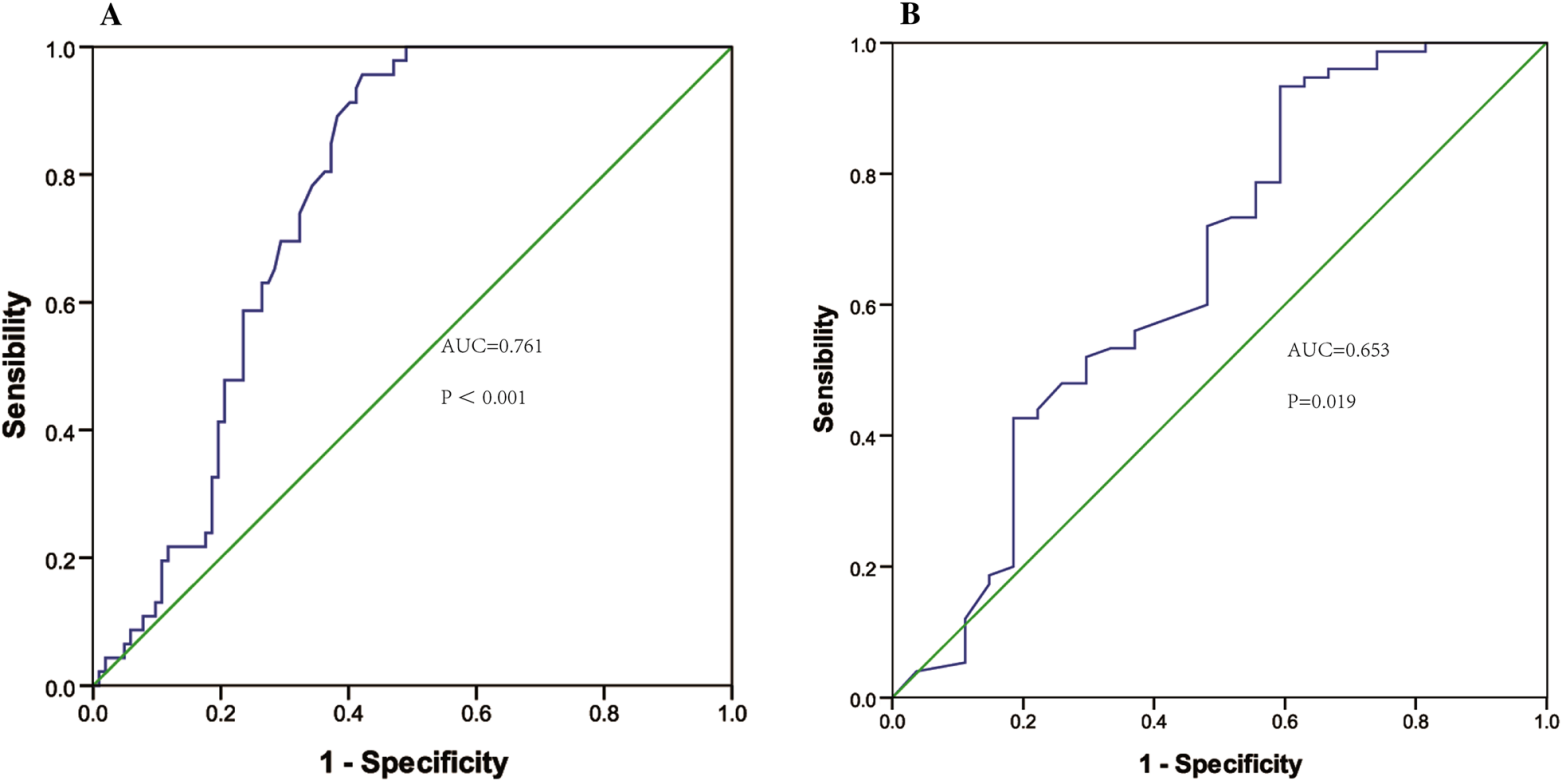
**A** ROC curve analysis for serum MFAP4 concentration and the prediction of the presence of AMI (STEMI and UNSTEMI). Area under the curve (AUC): 0.761 (95% confidential interval [CI] 0.687–0.835, *p* < 0.001). **B** ROC analysis between STEMI and UNSTEMI. Area under the curve (AUC): 0.653 (95% confidential interval [CI] 0.519–0.788, *p* = 0.019)

In order to differentiate AMI from UA, ROC was constructed and the area under the curve (AUC) of MFAP4 was 0.761 (*p* < 0.001, 95% CI 0.687–0.835). The optimal cut-off point of 0.117 ng/ml was selected with the higher sensitivity (96.7%) and specificity (57.8%) (Fig. 3A). Additionally, in differential diagnosis between STEMI and non-STEMI, ROC analysis showed that the AUC was 0.653 (*p* = 0.019, 95% CI 0.519–0.788) (Fig. 3B), indicating that MFAP4 may be a potential biomarker.

**Fig. 4:**
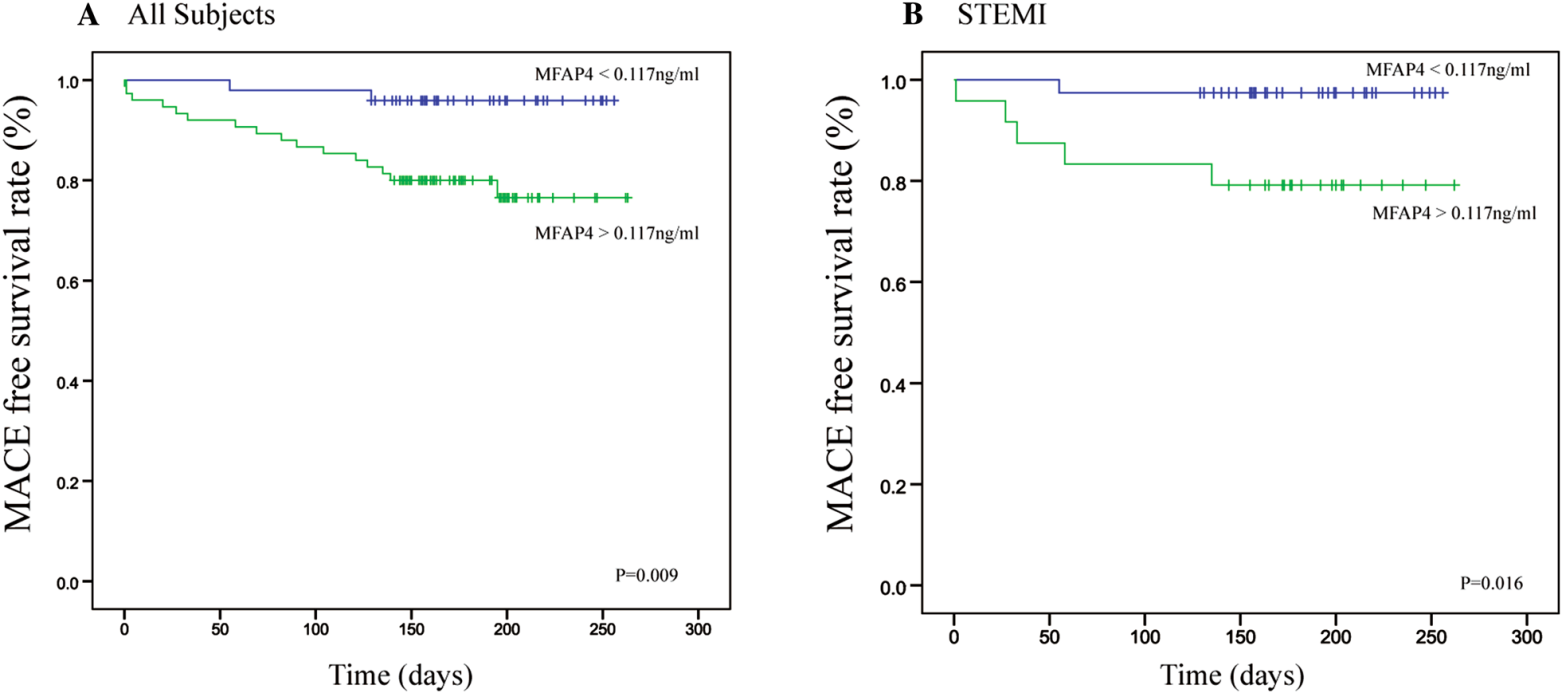
**A** Kaplan–Meier curve of MACE free survival time in all subjects. **B** Kaplan–Meier curves of MACE free survival time within the STEMI group. Patients were divided into low and high groups according to the cut-off value of sMFAP4

**Fig. 5:**
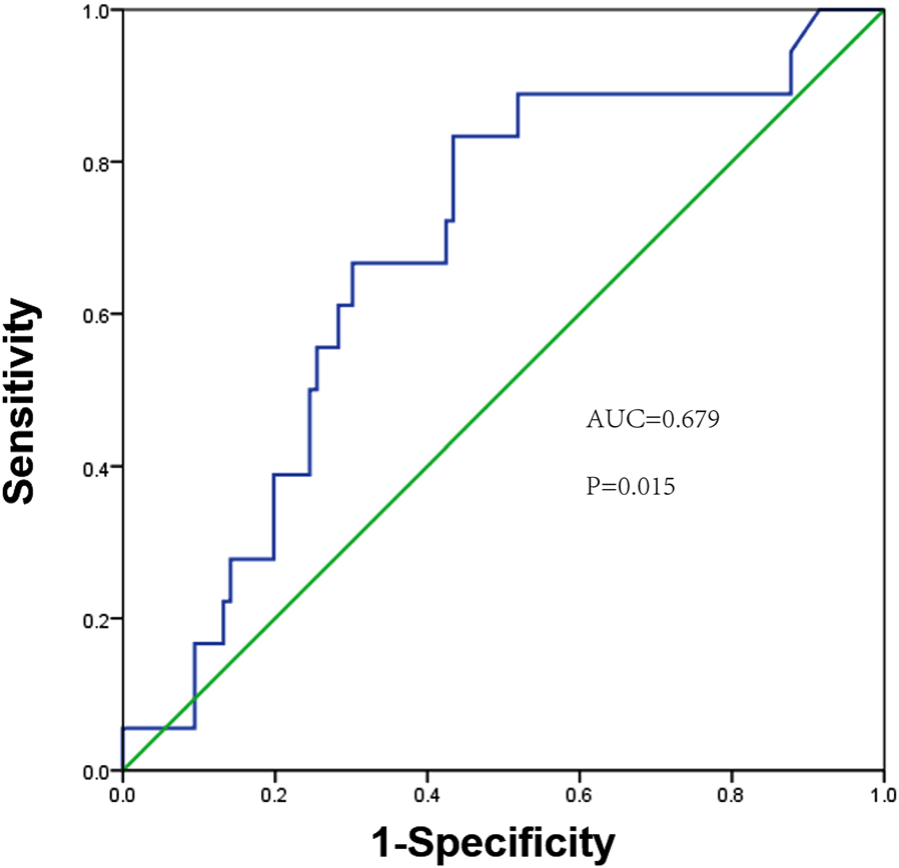
ROC curve analysis of baseline plasma MFAP4 for the prediction of MACE in ACS. Area under the curve (AUC): 0.679 (95% confidential interval [CI] 0.554–0.805, *p* = 0.015). MACE, major adverse cardiovascular events; ACS, acute coronary syndrome

According to the cut-off value (0.117 ng/ml), ACS patients were divided into two groups: the low-MFAP4 group (MFAP4 < 0.117 ng/ml, *n* = 60) and the high-MFAP4 group (MFAP4 ≥ 0.117 ng/ml, *n* = 88). The median follow-up was 165 (147, 199) days, and in Kaplan–Meier survival analysis, patients of lower MFAP4 levels had an increased event-free survival rate (*p* = 0.009) (Fig. 4A), and when this analysis was performed in the STEMI group, the same significant outcome was obtained (*p* = 0.016) (Fig. 4B). ROC curve analysis identified that the AUC for the baseline MFAP4 for the prediction of MACE in ACS was 0.679 (95% CI 0.554–0.805, *p* = 0.015) (Fig. 5).

## Discussion

This is the first study to date evaluating the association between circulating plasma MFAP4 levels and ACS and its clinical outcomes. In this study, we presented a significantly decreased MFAP4 concentration in STEMI, compared with non-STEMI and UA, which was correlated with coronary stenosis and myocardial injury markers, and to our surprise further demonstrated that higher MFAP4 levels prospectively predict the development of MACE in patients with ACS.

Human MFAP4 was mapped to the 17p11.2 chromosome and is first reported in Smith–Magenis syndrome [10]. It belongs to a member of the microfibrillar-associated proteins (MFAPs) family, and the majority of MFAPs are traditionally involved in the organization of elastic fibers by specifically binding to tropoelastin [4]. Similarly, MFAP4, ubiquitously distributed in extracellular matrix, was found to promote tropoelastin self-assembly, involved in proper elastic organization, and specifically bind fibrillin-1 and -2, as well as the elastin cross-linking amino acid desmosine [11]. MFAP4 mRNA relative transcript levels are highest in the heart, but the greatest abundance of MFAP4 is located in elastic fibers within blood vessels and surrounding connective tissue; cardiomyocytes do not contain MFAP4 [3].

Although its biological functions are not fully investigated, MFAP4 probably contributed to many disease conditions. As reported, in pulmonary physiology MFAP4 was involved via modulation of airway smooth muscle cells, participation in maintaining the integrity of the alveolar septa and interaction with lung surfactant protein D [12]. Since the presence of soluble desmosine is an indicator of elastic fiber degradation, plasma MFAP4 levels has been a potential indicator for chronic obstructive pulmonary disease (COPD) severity [13]. Also, MFAP4 has been proposed as markers of tumor progression and is linked to breast cancer [14], serious ovarian cancer [15] and neuroblastoma [16]. Additionally, initially identified as a biomarker of liver fibrosis, MFAP4 was subsequently confirmed its diagnostic capability in hepatitis C virus or alcohol-induced liver fibrosis [17]. Regarding artherosclerotic diseases, the expression of MFAP4 was reported to increase in vascular smooth muscle cells within large arteries, especially be activated and released following injury [2]. Previous basic studies on *Mfap4*-deficient mice have confirmed that MFAP4 participated in vascular smooth muscle hyperplasia and vascular remodeling in peripheral artery disease (PAD), and subsequent clinical research revealed that in symptomatic PAD patients upper tertile MFAP4 was significantly associated with cardiovascular death, but a surprisingly decreased risk of occlusion of reconstructed vessel [18]. Similarly, in our study, the baseline plasma MFAP4 level was negatively associated with coronary stenosis while increased MFAP4 levels prospectively predict the development of MACE in ACS (see Additional file 1).

A possible explanation of our present findings of negative relationship between MFAP4 levels and coronary stenosis in ACS may attribute extracellular matrix remodeling during plaque rupture. As one of the determinants of ACS severity [19], the integrity of the fibrous cap over plaque and its resistance to rupture depends critically on the content of extracellular matrix collagen and elastic fibers [20, 21]. Binding analysis revealed that MFAP4 specifically binds tropoelastin and fibrillin-1 and -2, as well as the elastin cross-linking amino acid desmosine. Site-directed mutagenesis disclosed residues Phe^241^ and Ser^203^ in MFAP4 as being crucial for type I collagen, elastin, and tropoelastin binding. Besides, the same study found that MFAP4 actively promotes tropoelastin self-assembly [11]. Thus, low MFAP4 levels are likely indicative of fibrous cap instability, which help explain why MFAP4 levels were relatively lower in STEMI compared with non-STEMI and UA. In agreement with our findings, Stakhneva E et al. found that MFAP4 levels increased only in stable atherosclerotic plaques in the fat deposition and fibrosis stages, but not in the fibrosis and calcification stages, nor in unstable atherosclerotic plaques of the necrotrophic-dystrophic type [22].

Contrary to the opposite trend between MFAP4 and coronary stenosis, our study found a significant decrease in asymptomatic survival in the high MFAP4 group, which may suggest that MFAP4 plays different roles in the disease process. As illustrated by Helle, MFAP4levels were particularly lower in patients with stable atherosclerotic disease compared with STEMI and non-STEMI [3]. With gradually aggravated myocardial ischemia, increased MFAP4 probably help alleviate vascular injury and subsequent atherosclerotic changes, and contribute to neovascularization of ischemic myocardium. Vascular smooth muscle cells (VSMCs) activation and phenotypic switching are critical for vascular remodeling wherein integrins are of particular importance [23]. MFAP4 serves as an integrin ligand localized to extracellular matrix fibers in the vascular wall, which has been found to regulate integrin αVβ3-induced VSMC proliferation and migration, and to accelerate neointimal hyperplasia after vascular injury [2]. Culture of isolated human saphenous veins for 14–21 days showed that MFAP4 was present in the vessel wall and that its deposition was induced in the area directly below the growing neointima [2], demonstrating that MFAP4 concentrations may parallel intimal neointima. We therefore suggest that MFAP4 may increase the risk of restenosis after coronary intervention, which explains why high levels of mfap4, a protective factor in ACS, was a predictor of MACE events in the present study. Further experiments are needed to monitor MFAP4 concentrations over longer cycles after the onset of ACS.

## Limitations

Several limitations of present study need to be considered. First, relatively small cohort and limited follow-up in this study limited further valuable information on the role of MFAP4 in ACS. Second, healthy controls were not included and other biomarkers were not sampled in this study. Third, there was no follow-up data on serial changes in MFAP4. Thus, a large-scale prospective with a longer duration of follow-up is need.

## Conclusion

The present study shows that MFAP4 levels were negatively correlated with the severity of coronary stenosis, and specifically low MFAP4 was an independent determinant of STEMI. Although further analysis shows higher MFAP4 levels prospectively predict the development of MACE, validating analyses of this relationship in larger cohort are required.

## Supplementary Information

**Additional file 1: Table S1. **Medication on admission and medication on discharge for the patient cohort.

## Data Availability

Data sharing is not applicable to this article as no datasets were generated or analyzed during the current study.

## Abbreviations

ACS: Acute coronary syndrome
AUC: Area under the curve
CKMB: Creatine kinase-MB
cTn: Cardiac troponins
LDL: Low-density lipoprotein cholesterol
MFAP4: Microfibrillar-associated proteins
MI: Myocardial infarction
non-STEMI: Non-ST-segment elevation myocardial infarction
STEMI: ST-segment elevation myocardial infarction
TC: Total cholesterol
UA: Unstable angina
