# Supplementary material for: Declined plasma microfibrillar-associated protein 4 levels in acute coronary syndrome
Source: Eur J Med Res. 2023 Jan 18;28:32. doi: 10.1186/s40001-023-01002-z (PMC9847181; doi:10.1186/s40001-023-01002-z)
Supplement: Supplementary file 1 — Additional file 1: Table S1. Medication on admission and medication on discharge for the patient cohort. [file 40001_2023_1002_MOESM1_ESM.docx]

| **Table S1.** Medication on admission and medication on discharge for the patient cohort | | | | | |
| --- | --- | --- | --- | --- | --- |
| **Clinical characteristics** | | **STEMI** | **UNSTEMI** | **UA** | **P-value** |
| **Total n=148** | | **n=75** | **n=27** | **n=46** |  |
| Medication  on  admission | ACEI/ARB[n(%)] | 14(18.7) | 4(14.8) | 17(37) | 0.035 |
|  | βRB[n(%)] | 6(8) | 3(11.1) | 7(15.2) | 0.438 |
|  | CCB[n(%)] | 14(18.7) | 12(44.4) | 18(39.1) | 0.01 |
|  | Diuretics[n(%)] | 2(2.7) | 2(7.4) | 1(2.2) | 0.48 |
|  | Nitrate[n(%)] | 1(1.3) | 0(0) | 1(0.7) | 1 |
|  | Aspirin[n(%)] | 0(0) | 0(0) | 3(6.5) | 0.034 |
|  | Statins[n(%)] | 10(13.3) | 5(18.5) | 13(28.3) | 0.126 |
|  | Ezetimibe[n(%)] | 0(0) | 0(0) | 1(2.2) | 0.493 |
|  |  |  |  |  |  |
| Medication  on  discharge | ACEI/ARB[n(%)] | 35(46.7) | 15(57.7) | 19(41.3) | 0.407 |
|  | βRB[n(%)] | 52(69.3) | 16(61.5) | 25(54.3) | 0.247 |
|  | CCB[n(%)] | 4(5.3) | 5(19.2) | 23(50) | ＜0.001 |
|  | Diuretics[n(%)] | 10(13.3) | 5(18.5) | 4(8.7) | 0.427 |
|  | Nitrate drugs[n(%)] | 18(24) | 3(11.5) | 19(41.3) | 0.016 |
|  | Aspirin[n(%)] | 71(94.7) | 26(100) | 45(97.8) | 0.578 |
|  | Clopidogrel[n(%)] | 54(72) | 20(76.9) | 32(69.6) | 0.799 |
|  | Ticagrelor[n(%)] | 19(25.3) | 5(19.2) | 4(8.7) | 0.078 |
|  | Statins[n(%)] | 69(92) | 24(92.3) | 44(95.7) | 0.75 |
|  | Ezetimibe[n(%)] | 59(79.7) | 20(76.9) | 11(23.9) | ＜0.001 |
